# Reverse epidemiology of obesity paradox: Fact or fiction?

**DOI:** 10.14814/phy2.70107

**Published:** 2024-10-29

**Authors:** Bellamkonda K. Kishore

**Affiliations:** ^1^ Division of Nephrology and Hypertension, Department of Internal Medicine University of Utah Health Salt Lake City Utah USA

**Keywords:** BMI paradox, cardio‐respiratory fitness, cardiovascular decompensation, lean diabetes, metabolically healthy obesity, non‐communicable diseases, obesity paradox

## Abstract

Obesity paradox refers to the clinical observation that when acute cardiovascular decompensation occurs, patients with obesity may have a survival benefit. This apparently runs counter to the epidemiology of obesity, which may increase the risk for non‐communicable diseases (NCDs). The scientific community is split on obesity paradox, with some supporting it, while others call it BMI paradox. This review: (a) defines the obesity paradox, and its proposed role in overall mortality in NCDs; (b) delineates evidence for and against obesity paradox; (c) presents the importance of using different indices of body mass to assess the risk in NCDs; (d) examines the role of metabolically healthy obesity in obesity paradox, and emerging importance of cardio‐respiratory fitness (CRF) as an independent predictor of CVD risk and all‐cause mortality in patients with/without obesity. Evidence suggests that the development of obesity and insulin resistance are influenced by genetic (or ethnic) make up and dietary habits (culture) of the individuals. Hence, this review presents lean diabetes, which has higher total CVD and non‐CVD mortality as compared to diabetics with obesity and the possibility of maternal factors programming cardiometabolic risk during fetal development, which may lead to a paradigm shift in our understanding of obesity.

## Graphical abstract

Obesity paradox is a clinical observation that when acute cardiovascular decompensation occurs, patients with obesity may have survival benefits. Development of insulin resistance, decrease in insulin secretion, and body fat distribution in obesity varies considerably based on ethnicity and dietary habits of people. Maternal factors may program fetal cardiovascular risk, which often leads to development lean diabetes, which has higher prevalence of complications and mortality than in obese diabetics. Cardio‐respiratory fitness (CRF) has emerged as an independent risk factor for death, irrespective of the obesity status of the subject. CRF may also influence mortality in obesity paradox.

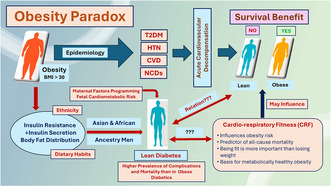

## 1 INTRODUCTION

**FIGURE 1:**
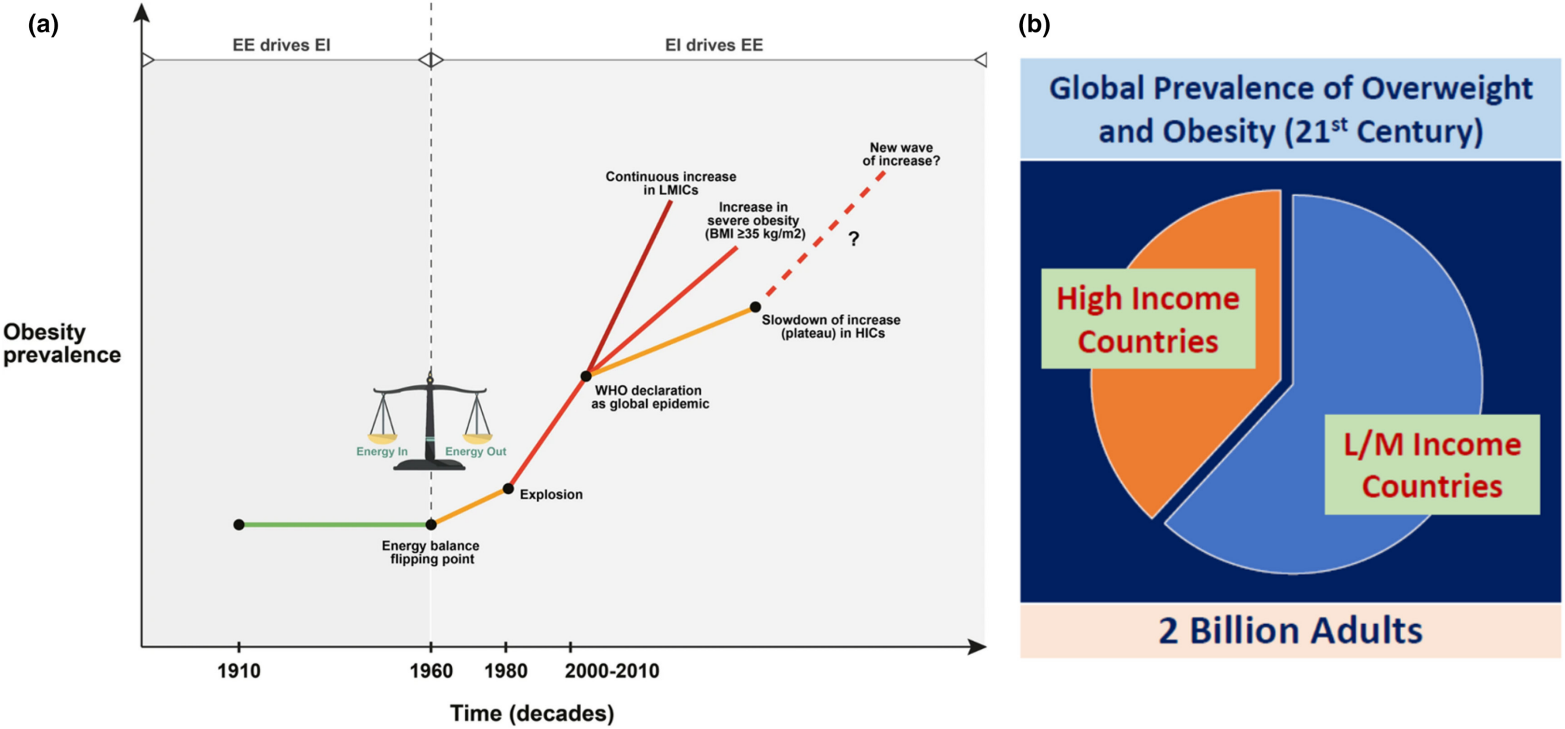
Prevalence of overweight and obesity in high income (HIC) and Low‐middle income (LMIC) countries. (a) A graphical summary of global obesity prevalence trends over decades of the 20th and 21st century. Reproduced from Koliaki et al. (2023) under creative commons CC BY license. (b) Proportion of global prevalence of overweight and obesity in twentieth century in high‐income and low‐middle income countries, based on several reports.

With over 2 billion people being overweight (Body mass index or BMI >25 Kg/m^2^), obesity is a global problem (Sørensen et al., 2022). Of these about 600 million people are having obesity (BMI >30 Kg/m^2^). If we apply the Asia‐Pacific classification of obesity recommended by the World Health Organization (n.d.) (WHO—Obesity), the actual number of subjects with obesity will be much higher (Table 1). Most of these people are living in emerging economies (BRICS countries) and in rapidly developing countries of Asia, Africa, and South America (Figure 1; Jakovljevic & Milovanovic, 2015; Koliaki et al., 2023). Incidentally, these are also the regions where there is steep rise in the prevalence of non‐communicable diseases (NCDs), such as diabetes mellitus, hypertension, cardiovascular and cerebrovascular disorders with acute events, chronic kidney disease, and other NCDs (Jakovljevic & Milovanovic, 2015; Ndubuisi, 2021; Remais et al., 2013). Thus, emerging economies and the developing world are bearing a disproportionately higher burden in dealing with obesity and its consequences. A closer examination of this disparity reveals many variables starting from genetic make up to dietary and lifestyle habits, to environmental factors (Hu, 2011; van Vliet‐Ostaptchouk et al., 2012; Bhurosy & Jeewon, 2014; Albuquerque et al., 2017; Kang et al., 2021). But most studies on obesity and its complications as well as their management are performed on people in developed countries, with different genetic makeup, dietary habits, and lifestyle. It is time to examine whether we are understanding the complex subject of obesity in its totality or in titbits which are easier to do. In this context, the controversial subject “obesity paradox” offers an opportunity to re‐examine our position on obesity and contemplate whether we are getting it right or oversimplifying a complex subject for our convenience to handle or deal with.

**TABLE 1:** Cut points for overweight and obesity indicators used for South Asians versus Westerners.

Indicators	South Asians	Westerners
Body mass index (BMI) kg/m^2^		
Underweight	<18.5	<18.5
Normal Weight	18.8–22.9	18.5–24.9
Overweight	23.0–24.9	25.0–29.9
Obese I	25.0–29.9	30.0–34.9
Obese II	30.0–34.9	35.0–39.9
Obese III	35.0–39.9	>40.0
Waist circumference (cm)		
Men	≥90	≥102
Women	≥80	≥88
Waist hip ratio		
Men	≥0.9	≥0.9
Women	≥0.8	≥0.8
Body fat percentage		
Men	≥20%	≥25%
Women	≥33%	≥35%

Using BMI as a benchmark, many experimental, clinical, and epidemiological studies linked overweight and obesity for the development of a variety of NCDs, such as type‐2 diabetes mellitus (T2DM), hypertension, and diseases of the heart, liver, lung, and the kidney among others (Akhter et al., 2021; Ejigu & Tiruneh, 2023; Felisbino‐Mendes et al., 2020; Fuentes et al., 2023; Kilpi et al., 2014; Kivimaki et al., 2022; Lobstein & Brinsden, 2014; Nyberg et al., 2018; Webber et al., 2012; Zatońska et al., 2021; Zhang et al., 2024). Obesity is also a determining factor for longevity (Donini et al., 2012; Lenz et al., 2009; Lung et al., 2019; Peeters et al., 2003; Solomon & Manson, 1997; Tam et al., 2020). Currently, NCDs account for 41 million deaths per year globally (74% of all deaths). Of these 77% of deaths occur in low‐ and middle‐income countries (WHO–NCDs). It is projected that by the year 2030, NCDs will cause 52 million deaths. According to the WHO, about 35.8 million (2.3%) of global DALYs (disability adjusted life years) are lost by overweight or obesity (WHO–NCDs). In June 2023, the American Medical Association officially recognized *obesity as a disease state with multiple pathophysiological aspects requiring a range of interventions to advance obesity treatment and prevention*. AMA Policy # H‐440‐842 (n.d.). According to an extensive analysis made by McKinsey Global Institute, in 2014 four preventable causes accounted for the loss of $7.6 trillion globally (9.51% of global GDP in 2014). These are smoking ($2.1 trillion), armed violence, war, and terrorism ($2.1 trillion), obesity ($2.0 trillion), and alcoholism ($1.4 trillion) (Dobbs et al., 2014). Hence, logically reducing the global burden of obesity will eventually result in lower number of deaths due to NCDs with substantial savings in healthcare costs. However, it is easier said than done. Because the accumulated knowledge on obesity and its health consequences in various racial or ethnic groups strongly suggests that one size fits all formulas may not work. For example, the primary metabolic abnormality in overweight or obesity—*development of insulin resistance*—is highly variable among different ethnic groups in a gender‐dependent manner (reviewed in Kishore, 2022). Storing fat is an evolutionary protective mechanism for survival (Genné‐Bacon, 2014; Speakman & Elmquist, 2022; Wells, 2012). Optimum levels of adipose tissue act like a fuel injection system of the internal combustion engine, and thus regulate whole body energy and glucose homeostasis. The “set‐point theory” postulates that human body has a predetermined weight or fat mass set to a range. Compensatory physiological mechanisms and redundant pathways maintain that set‐point (Ganipisetti & Bollimunta, 2023). By secreting bioactive molecules or hormones such as leptin and adiponectin, and other substances, adipose tissue regulates energy for growth and efficient function of the immune system.

In the above context, obesity paradox offers an opportunity to re‐evaluate and understand the complex subject of obesity with the sole intention of *knowing enough to know whether we are right or wrong*. Obesity paradox refers to the clinical observation that when acute cardiovascular decompensation occurs, obese patients may have a survival benefit. In recent years several review articles have been published on the obesity paradox (Assaf & Antoun, 2021; Braun et al., 2015; Donini et al., 2020; Dramé & Godaert, 2023; Liu et al., 2022; Simati et al., 2023). In addition to presenting the salient features in those review articles, this review provides additional information. The review presents unbiased facts with evidence on all aspects of obesity paradox. This review also attempts to provide a comprehensive landscape of obesity by dealing with different types of obesity vis‐à‐vis cardiometabolic risk, lean diabetes (LD), and potential maternal and infant nutritional insults in programming obese phenotype and cardiometabolic risk. Finally, this review article deals with obesity paradox and a related condition, LD on the same platform. It is the expectation of the author that this review will stimulate a new wave of thinking and approach to tackle the global problem of obesity. Although this review may not offer answers to all the questions we may have or give a final verdict on “fact or fiction” this is a small step in the right direction.

## 2 HISTORICAL ASPECTS

**FIGURE 2:**
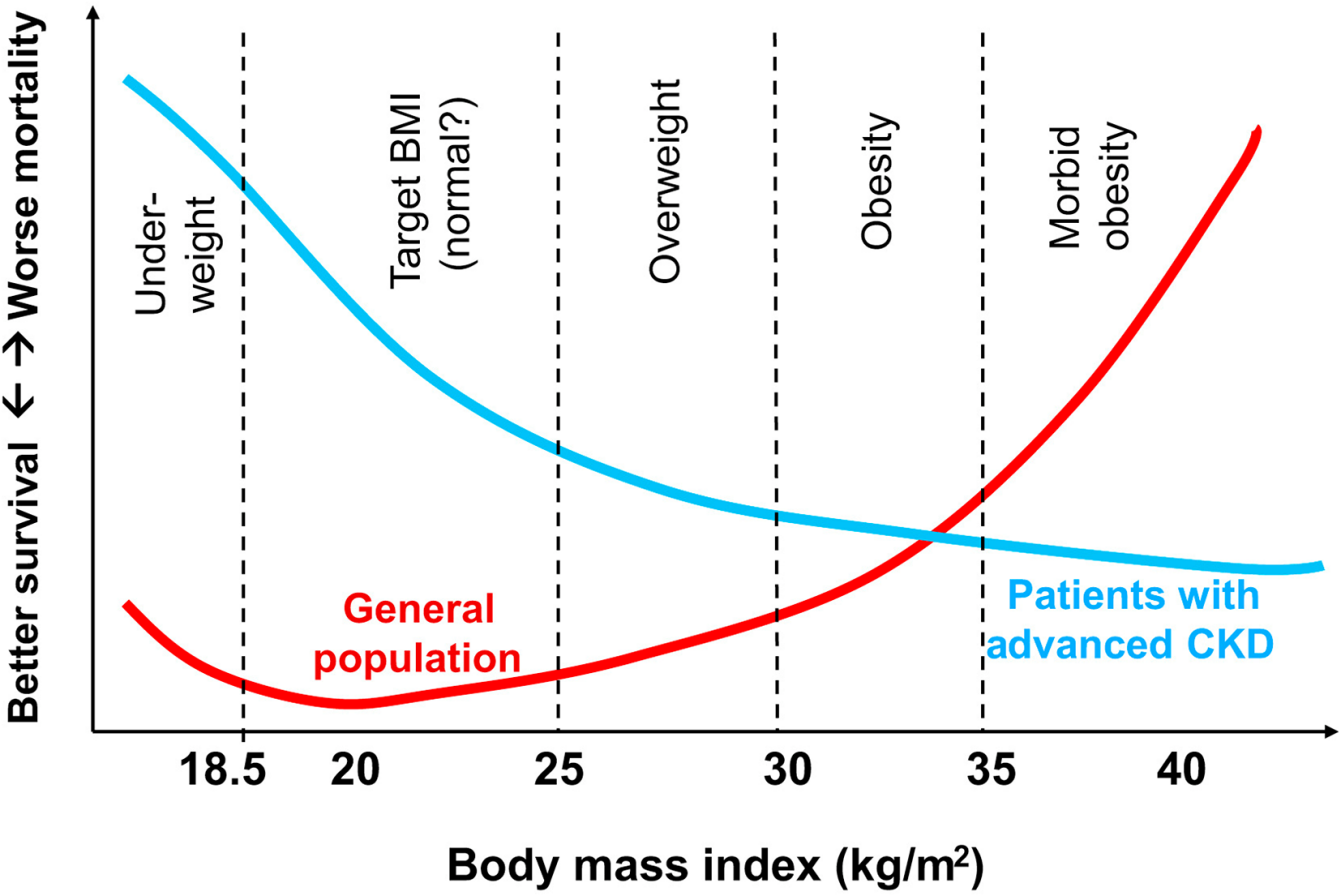
Reverse association of BMI and survival in patients with advanced chronic kidney disease (CKD) as compared to the general population. Reproduced from Kalantar‐Zadeh et al. (2017) under creative commons CC‐BY‐NC‐ND.

Dr. Kalantar‐Zedah first proposed the term “reverse epidemiology” in the journal *Kidney International* in 2003, and in the *Journal of the American College of Cardiology* in 2004 (Kalantar‐Zadeh et al., 2003, 2004). He reported that conventional risk factors for cardiovascular diseases (CVD) in general population, such as high body mass, serum cholesterol, and blood pressure, were often found to be protective and associated with greater survival among patients on maintenance hemodialysis (Figure 2). At that time, it was considered a contradiction to prevailing medical concepts of prevention of CVD. Although first described in dialysis patients, subsequently obesity paradox was found in those with heart failure, myocardial infarction, acute coronary syndrome, chronic obstructive pulmonary disease (COPD), rheumatoid arthritis, and older residents in nursing homes (Dramé & Godaert, 2023; Eitmann et al., 2024; Escalante et al., 2005; Kalantar‐Zadeh et al., 2017; Lainscak et al., 2012; Liu et al., 2022). It should be noted that the obesity paradox does not contradict the epidemiological data that obesity predisposes people to the development of NCDs. Obesity paradox states that once people develop NCDs, being obese offers a protective mechanism against mortality at times of cardiovascular decompensation.

## 3 CRITIQUES OF OBESITY PARADOX

As expected, not everyone agrees about the possibility of obesity protecting patients with chronic NCDs. The critiques argue: (i) body fat might offer protection to survive during periods of low nutrition; (ii) the non‐obese population at risk are those who have lost weight because of more severe illness; (iii) people with obesity are diagnosed early with NCDs; (iv) BMI is poor indicator of body fat; (v) BMI cut‐offs are not appropriate, and (vi) the observed obesity paradox is due to Collider Stratification Bias. It is possible to verify or eliminate the first three critiques in carefully controlled studies. But the argument against BMI is counterintuitive, as the same BMI is the benchmark in epidemiological studies which revealed that obesity predisposes to NCDs. The issue of Collider Stratification Bias is explained below.

**FIGURE 3:**
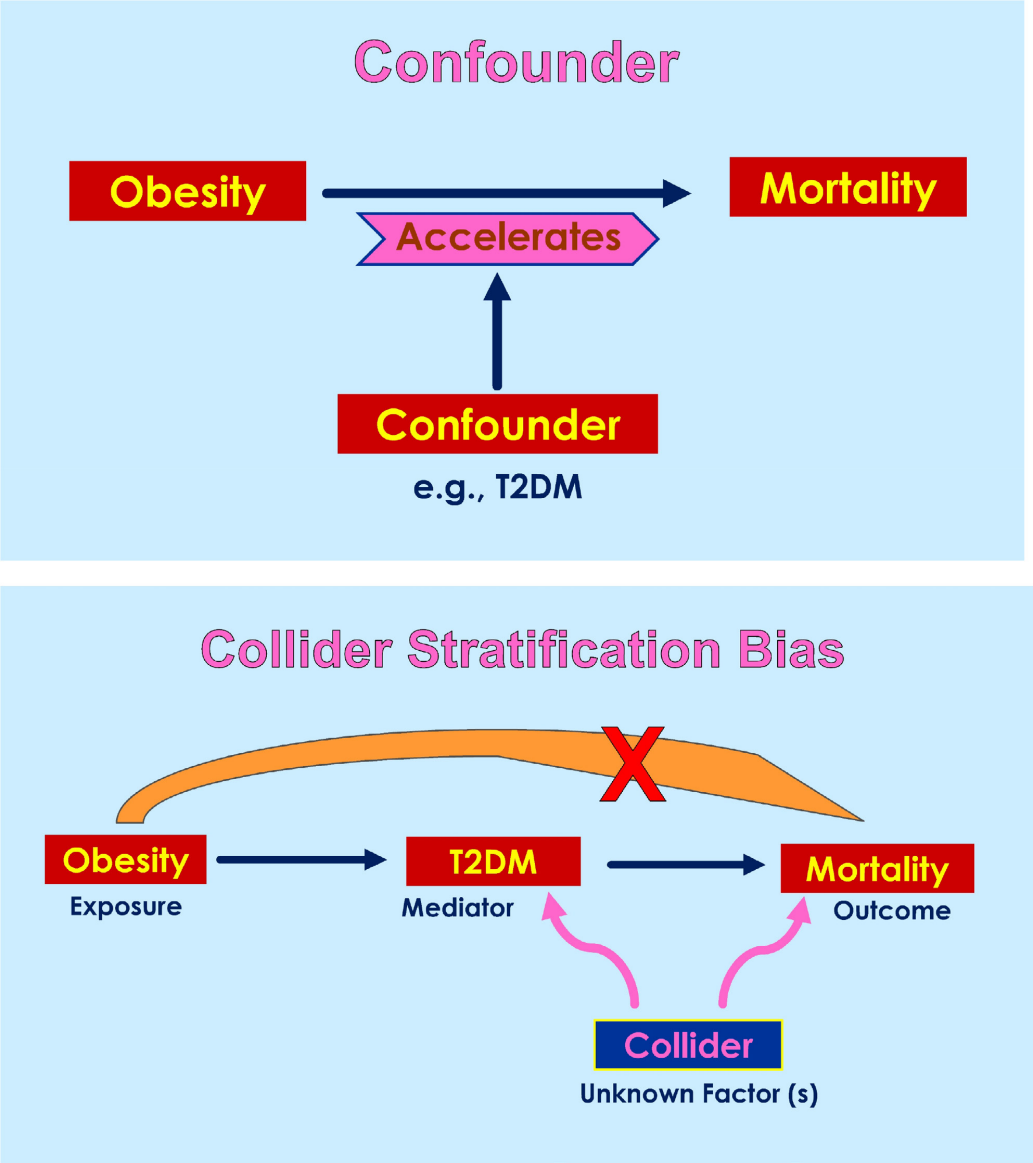
Confounder versus Collider Stratification Bias. Unlike a confounding factor, a collider may introduce a spurious association between the cause and effect, and thus negatively affect the outcome, that is, in this case obesity may protect against mortality, instead of causing mortality.

Collider for a pair of variables is often a third variable influenced by both. Unlike a confounding factor, a collider may introduce a spurious association between the cause and effect, and thus negatively affect the outcome, that is, in this case being obese may protect against mortality, instead of causing mortality. Thus, a collider differs from a confounder (Figure 3). There are good reviews on collider bias with examples for the readers who would like to know more about on this subject (Day et al., 2016; Tönnies et al., 2022). However, it has been shown that collider bias alone cannot fully explain obesity paradox. Collider bias may explain only a small discrepancy between the association and the casual effect observed (Sperrin et al., 2016). Collider bias must also be strong enough to have a consistent association reversing the casual effect (Glymour & Vittinghoff, 2014). Last, when the population is unselected, collider bias may not work (Flegal et al., 2013). We know that is not the case with obesity.

## 4 OBESITY PARADOX VERSUS BMI PARADOX

**FIGURE 4:**
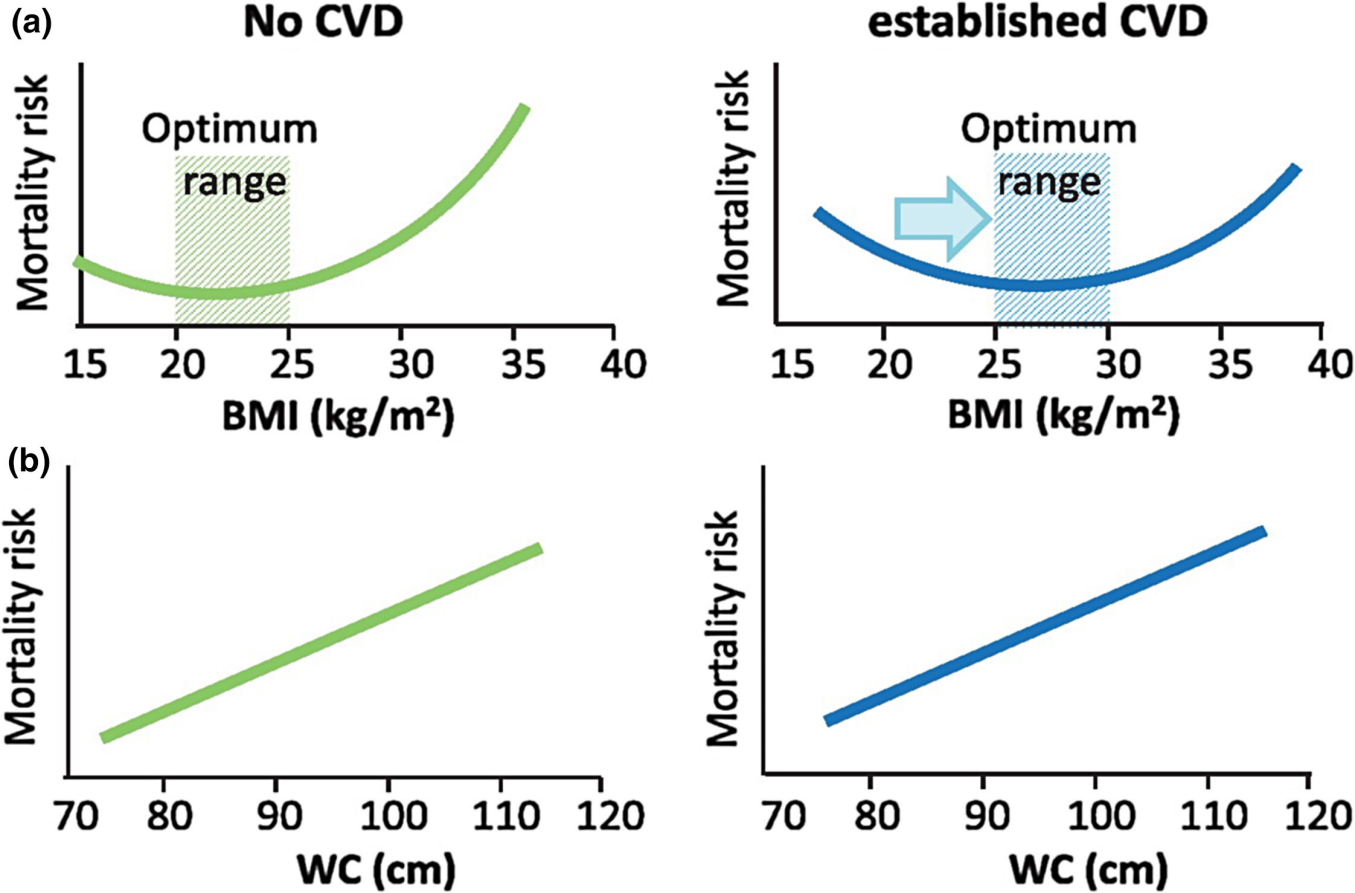
Association among BMI, waist circumference, and mortality risk. When BMI is used as the benchmark of obesity, the mortality risk takes a J‐ or U‐shaped curve in patients with CVD or without CVD, respectively. The optimum range of BMI also shifts from 20–25 to 35–30 Kg/m^2^. However, when the waist circumference (WC) is taken as the parameter, the association with mortality risk is linear in both groups of subjects. Reproduced with permission from Antonopoulos & Tousoulis. (2017). Oxford University Press License No. 5812090316656 dated June 18, 2024.

If we use BMI as the benchmark, then we will observe a J‐shaped relation between BMI and mortality risk in subjects with no CVD, with optimum BMI being between 20 and 25 Kg/m^2^. But in subjects with established CVD, this relation between BMI and mortality risk becomes U‐shaped with the optimum range of BMI shifting to the right, 25–30 Kg/m^2^. However, this phenomenon could not be seen if we plot waist circumference (WC in cm) instead of BMI in the same population (Figure 4) (Antonopoulos & Tousoulis, 2017). Based on this observation critiques say what we see is BMI paradox, not obesity paradox. However, we knew that abdominal or visceral adiposity leads to deleterious metabolic disturbances, and subcutaneous fat accumulation has a benign effect on cardiometabolic risk (Blüher, 2020; Liu et al., 2011; Neeland et al., 2013; Sam, 2018). Several anthropometric indices independent of obesity paradox have been proposed (Table 2; Sommer et al., 2020; Wu et al., 2021; Gažarová et al., 2022; Li et al., 2024). These can be used in routine clinical practice. Sophisticated imaging analysis gives better information about distribution of fat depots in the body (Table 2) but are not usable routinely in the clinics.

**TABLE 2:** Assessment of overweight and obesity independent of BMI.

Clinically usable anthropometric indices independent of BMI
Waist circumference (WC)
Waist‐to‐hip ratio (WHR)
Waist‐to‐height ratio (WHtR)
Waist‐to‐hip‐to‐height ratio (WHHR)
Increased Body Fat Percent
Imaging indices for information on fat distribution (Not usable routinely)
Volumetric analysis of body fat depots (CT or MRI)
Intramuscular fat accumulation (Independent risk factor for CVD)
Ectopic fat accumulation (Bone marrow or liver)

## 5 CRF INFLUENCES OBESITY RISK

Cardio‐respiratory fitness (CRF) is the measure of the ability of circulatory and respiratory systems to supply oxygen to skeletal muscle mitochondria for energy production needed during sustained physical activity (Carrad et al., 2022; Franklin et al., 2023; Raghuveer et al., 2020). In 2016 the American Heart Association published an official document advocating that CRF, quantified as VO_2_ max/peak is a clinical vital sign, and advised that it should be routinely assessed as part of clinical practice (Ross et al., 2016). Meta‐analysis showed that CRF is a quantitative predictor of all‐cause mortality and cardiovascular events in healthy men and women (Ezzatvar et al., 2021; Fosstveit et al., 2024; Jiménez‐Pavón et al., 2019; Kodama et al., 2009; Lang et al., 2024). A cross‐sectional study from Germany showed that higher CRF is strongly associated with lower cardiovascular risk factors in firefighters, who suffer cardiovascular events on duty (Strauss et al., 2021). Recent studies also confirmed the relationship between CRF, cardiovascular risk factors, atherosclerosis, and morbidity and mortality (Chu et al., 2020; Lang et al., 2024).

Studies aimed at understanding the relationship between CRF, obesity, body composition, cardiometabolic risk and all‐cause mortality, and obesity paradox brought out interesting observations. For instance, examination of leanness and the hazards of obesity vis‐à‐vis CRF revealed that the health benefits of leanness are limited to fit subjects, and being fit may reduce the hazards of obesity and improve the quality of life (Blair et al., 1996; Flesaker et al., 2021; Lee et al., 1999). A cohort study involving 1 million men provided evidence for association between low levels of CRF and obesity with later risk of chronic disability due to CVD. The study suggested that preventive action may begin at young ages and include promotion of CRF and healthy body weight (Henriksson et al., 2020 Eur Heart J). A prospective observational study examined the relationship between low CRF, and mortality in subjects with normal weight, overweight and obesity, and reported that low CRF was a strong and independent predictor of CVD and all‐cause mortality, and of comparable importance with diabetes mellitus and other CVD risk factors (Wei et al., 1999 JAMA). A prospective study in women revealed that low CRF and higher BMI were independently associated with incident T2DM, with the protective effect of CRF seen in individuals who were overweight or obese (Sui et al., 2008). Even a moderate intensity exercise training improved CRF in women (Branch et al., 2000).

Thus, being fit is more important than losing weight in terms of lowering CVD mortality risk. Unfit subjects with obesity have almost two‐fold higher CVD risk compared to obese, but fit individuals. Subjects who are fit, but obese (fat but fit) have lower CVD risk compared to normal weight, but unfit individuals, as assessed by relative risk of all‐cause mortality by CRF levels (Hainer et al., 2009). So, BMI alone used as benchmark of obesity cannot identify the CVD risk.

## 6 METABOLICALLY HEALTHY OBESITY

**FIGURE 5:**
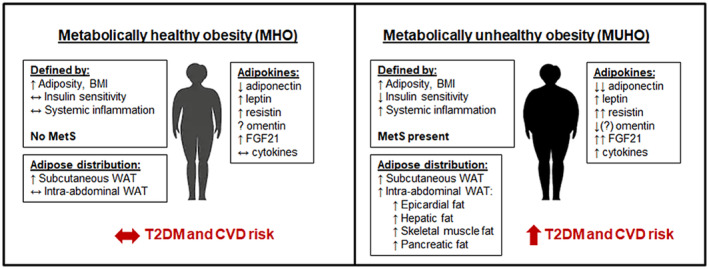
Metabolically healthy obesity (MHO) versus metabolically unhealthy obesity (MUHO). Note the differences between the two groups in insulin sensitivity, systemic inflammation, distribution of adipose tissue, and levels of adipokines. Figure reproduced from Chait & den Hartigh. (2020), under Creative Commons CC BY 4.0 Attribution 4.0 International.

**FIGURE 6:**
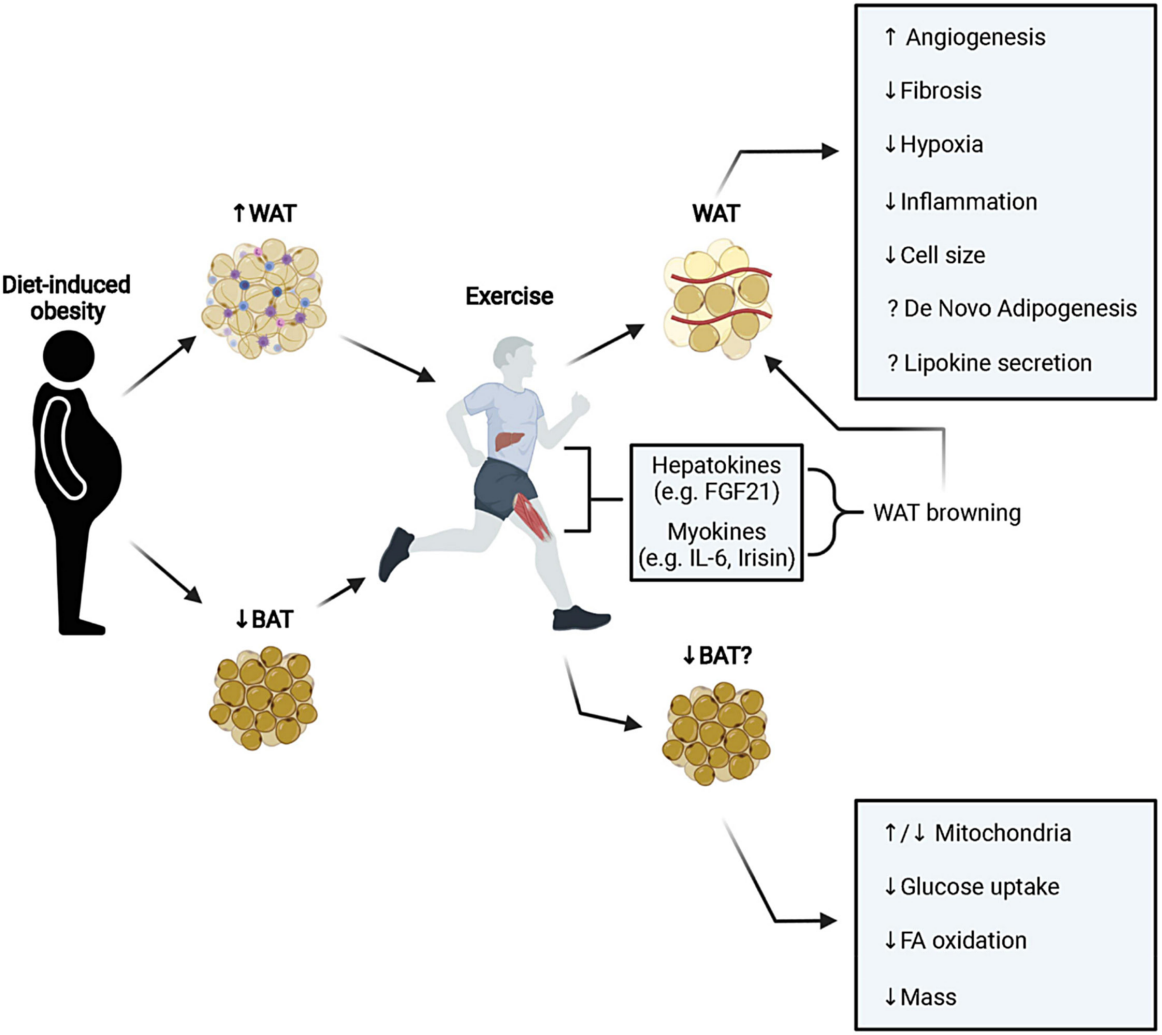
Remodeling of white and brown adipose tissues by exercise in subjects with diet‐induced obesity. As shown, in both types of adipose tissue, exercise has significant effect in modulating structure and function at the cellular and molecular levels. BAT, brown adipose tissue. WAT, white adipose tissue; FA, fatty acid; FGF21, fibroblast growth factor 21; IL‐6, interleukin‐6. Reproduced from Garriston & Boudina, (2021), under the creative commons attribution license (CC‐BY).

Recently, the concept that metabolically healthy obesity (MHO) phenotype exists has gained ground. MHO is defined as subjects with BMI of 30.0 devoid of signs of metabolic disorders, such as high blood pressure, high fasting blood glucose, low high‐density lipoprotein cholesterol, or high triglycerides. Wang et al. (2023) reported that among the 20,430 participants in 10 National Health and Nutrition Examination Survey (NHANES) cycles between 1999–2018 and 2017–2018, about 10.6%–15.0% US adults exhibited MHO phenotype. Figure 5 shows the physical, physiological and molecular characteristics of MHO versus metabolically unhealthy obesity (MUHO) (Chait & den Hartigh, 2020). Although MHO appears to be in a transient state, the fact it can be promoted by exercise is gaining ground (Ortega et al., 2018; Su et al., 2022; Tsatsoulis & Paschou, 2020). There is evidence that conversion between metabolically healthy and unhealthy obesity is possible from midlife to late life in all BMI categories (Ler et al., 2024), thus making this phenomenon worth probing further. Several studies documented the beneficial effects of regular exercise on regulation of the function of white and brown fat depots (Dewal & Stanford, 2019; Garriston & Boudina, 2021; Honkala et al., 2020; Jin et al., 2024; Lee et al., 2019; Stroh & Stanford, 2023; Vissers et al., 2013). Figure 6 shows how exercise remodels white and brown adipose tissue in individuals with diet‐induced obesity (Garriston & Boudina, 2021). Excellent review articles are available on the remodeling of white and brown adipose tissue and its role in energy metabolism, pathophysiology of metabolic disorders, and therapeutic drug development (Auger & Kajimura, 2023; Choe et al., 2016; Magro & Dias, 2024; Sakaguchi, 2024).

Conversely, there are subjects with metabolic obesity but with normal weight, the Metabolically obese, normal weight (MONW). The prevalence of MONW varies from 5%–45% depending on social and demographic factors as well as in defining the parameters (Conus et al., 2007). The MONW phenotype is associated with high prevalence of cardiometabolic dysregulation, metabolic syndrome, and cardiovascular risk factors (Table 3). In women, MONW is independently associated with increased risk of cardiometabolic mortality (Romero‐Corral & Somers, 2010). Table 4 lists the cardiometabolic abnormalities to be evaluated in all types of subjects with obesity to know which category they belong to (Wildman et al., 2008). To be considered as MONW phenotype, the subject should have BMI <25 kg/m^2^ and two or more cardiometabolic abnormalities.

**TABLE 3:** Metabolic characteristics of MONW and MHNW individuals.

Parameter	MONW	MHNW
Body mass index	Low	Low
Visceral fat	High	Low
Fat mass (as % of body mass)	High	Low
Lean body mass	Low	High
Insulin sensitivity	Low	High
Liver fat	High	Low
Serum triglycerides	High	Low

**TABLE 4:** Cardiometabolic abnormalities to be considered in patients for risk assessment.

	Cardiometabolic abnormalities to be considered in patients
1	High blood pressure ≥130/85 mm Hg or use of antihypertensive medications
2	High fasting serum triglyceride levels ≥150 mg/dL
3	Low HDL cholesterol level <40 mg/dL (men) or <50 mg/dL (women) or use of statins
4	High blood glucose levels ≥100 mg/dL (fasting) or use of anti‐diabetic medications
5	Insulin resistance: HOMA‐IR >5.13 (90th percentile)
6	Systemic inflammation: hsCRP level >0.1 mg/L (90th percentile)

## 7 LEAN DIABETES

### 7.1 Epidemiology and pathophysiology of lean diabetes

Although obesity is considered as the driver of T2DM, it is now recognized that a significant proportion of patients with diabetes are not obese, leading to the term Lean Diabetes (LD). LD is also known as Atypical Diabetes, Malnutrition‐related Diabetes, Tropical Diabetes, and by other names. LD does not meet the classification of T2DM given by the American Diabetes Association/World Health Organization (n.d.). In fact, as described below, LD may be a hybrid of T1DM and T2DM. Epidemiologically, LD is prevalent in men of Asian or African ancestry, often with history of childhood nutritional insults (Faraz et al., 2021; Kibirige et al., 2022). Nutritional insults in general refer to undernutrition or overnutrition or deficiency of certain components of the diet, such as micronutrients (Hsu & Tain, 2019). The prevalence of LD is also rising rapidly in the United States. Over a five‐year period (2015–2020), there was a 17.8% increase in LD in adults as compared to 2.1% increase in diabetes among people with overweight or obesity. The increase in the prevalence of LD in the United States is attributed to larger increases among women and people of color (Adesoba & Brown, 2023).

Typically, LD has an early age of onset. LD patients do not develop ketosis on withdrawal of insulin, but they might have higher total CVD and non‐CVD mortality vs. obese diabetics (reviewed in: George et al., 2015; Olaogun et al., 2020; Kishore, 2022; Salvatore et al., 2023). In addition, risk of hypoglycemia, and death is higher in LD patients. Morphologically, large adipocytes can be found in Asian LD patients associated with low levels of adiponectin and fatty acid breakdown, that age faster (cellular senescence). Thus, LD adipocytes switch from “fat storage” to “fat spillage”, negatively affecting CV system. LD patients also have higher HbA1c, fasting and post‐prandial blood glucose levels as compared to obese diabetics (reviewed in: Faraz et al., 2021; Kishore, 2022; Kibirige et al., 2022). Furthermore, microvascular complications of diabetes (e.g., retinopathy), nephropathy, and neuropathy are more common among male LD patients. The prevalence of LD is steadily increasing among Asians, especially in South Asia. In a prospective study sponsored by the Indian Council of Medical Research (ICMR), involving nine centers in India, the prevalence of LD varied from 11% to 25% (Das, 1993, 1994). The leanness in patients of this ICMR study persisted even after 5 years of follow up period. Thus, it seems leanness was the inherent characteristic of these individuals.

### 7.2 Ethnic differences in insulin sensitivity, insulin secretion, and body fat distribution

Several studies documented significant and clear ethnic differences in insulin sensitivity, insulin secretion, and body fat distribution, which incidentally correlates with the manifestation of LD with increased risk for developing CVD in certain ethnic populations. For instance, healthy Asian Indians have significantly greater abdominal and visceral fat, and insulin resistance compared to age and BMI matched Caucasians (Chandalia et al., 2007; Raji et al., 2001). Similarly, genetic background of Africans and East Asians makes them more and differentially susceptible to diabetes than Caucasians (Kodama et al., 2013). Interestingly, lean, non‐diabetic Asian Indians have decreased insulin sensitivity and insulin clearance and raised leptin levels compared to Caucasians and Chinese subjects (Liew et al., 2003). In fact, among lean Asians, Chinese are the most insulin sensitive whereas Asian Indians are the least insulin sensitive (Tan et al., 2015). These basic ethnic differences are directly reflected in the high prevalence of diabetes in South Asians or Asian Indians, as compared to Chinese, Caucasians and Blacks, irrespective of where they are living (Gujral et al., 2013; Kanaya et al., 2014; Ma & Chan, 2013; Narayan & Kanaya, 2020; Narayan et al., 2021). These studies underscore the importance of ethnicity while studying obesity and diabetes, as well as while treating these conditions in the clinics. In this respect, perhaps, obesity and insulin sensitivity, or resistance stand out as compared to other NCDs.

### 7.3 Evolutionary basis for low lean mass in Asian Indians

**FIGURE 7:**
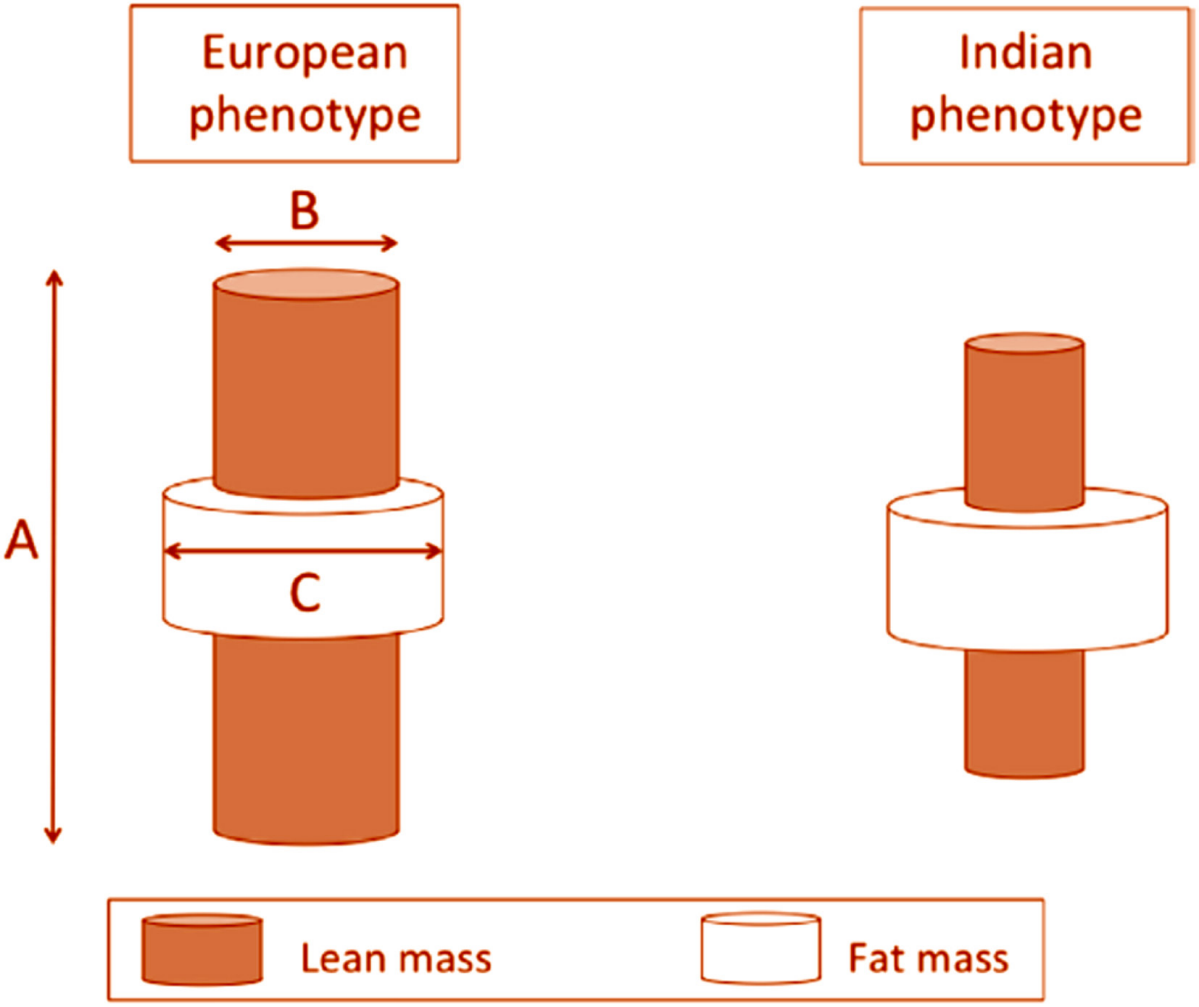
For a given Level of BMI, Asian Indians have higher proportion of fat mass in their body as compared to Westerners. The length (a) and cross‐sectional area (b) of an internal cylinder of lean mass is considered a marker for metabolic capacity, whereas the volume of the external cylinder of fat mass (c) is considered as a marker of metabolic load. Reproduced from Wells et al. (2016), under creative commons attribution 4.0 International CC‐BY 4.0.

**FIGURE 8:**
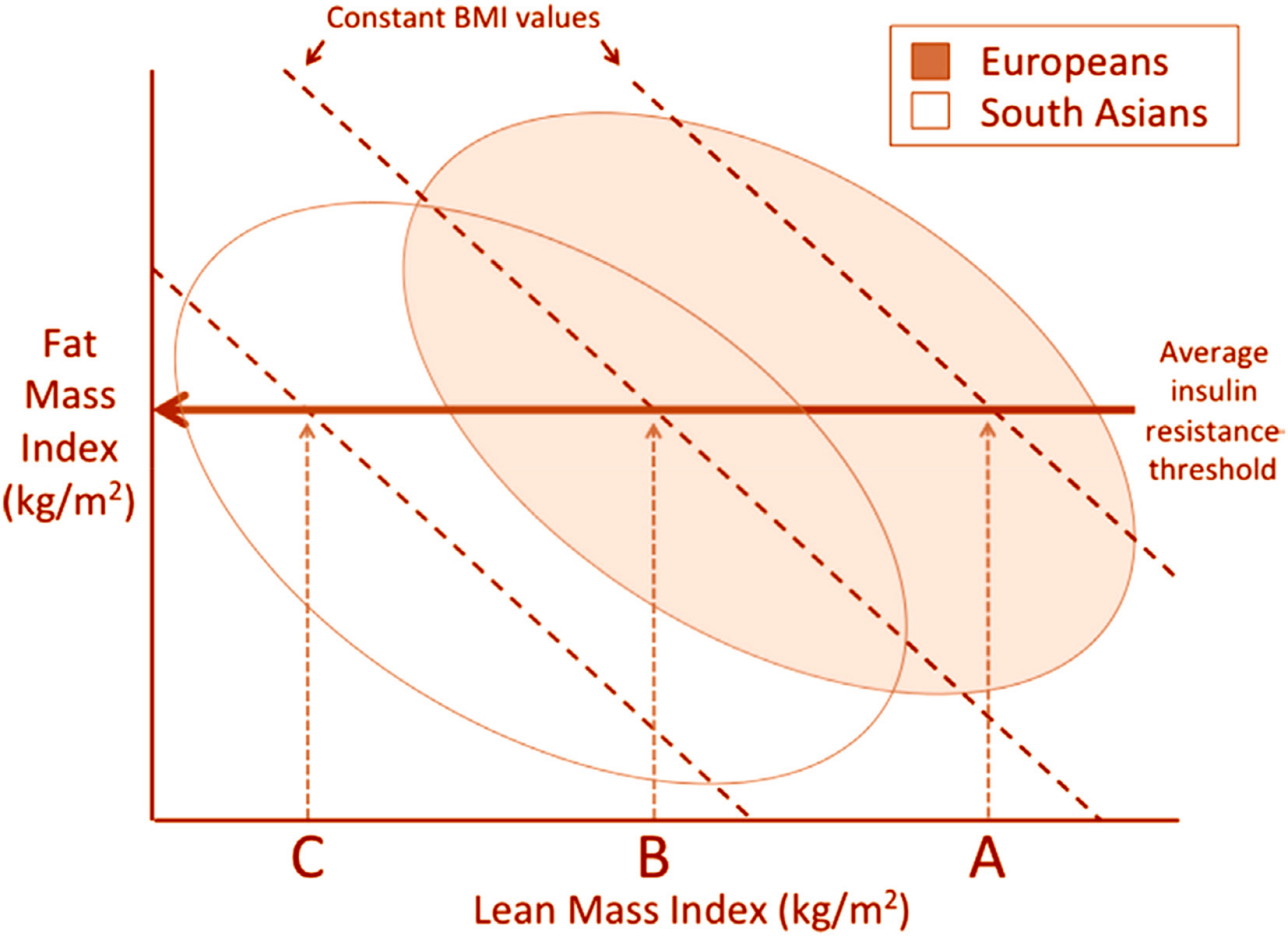
Plot of fat mass index (Fat mass divided by height squared) and lean mass index (Lean mass divided by height squared) in Europeans versus South Asians. Above a certain threshold of fat mass index, insulin resistance develops. Since Asians have lower lean mass, they develop insulin resistance at lower levels of BMI compared to Europeans. Reproduced from Wells et al. (2016) under creative commons attribution 4.0 International CC‐BY 4.0.

As illustrated in Figures 7 and 8, modern‐day South Asians have lower lean mass (organ and muscle mass) relative to those in Europe or Americas (Rush et al., 2009), which makes them prone to develop metabolic disorders at a lower BMI than other populations. People living in the Indian subcontinent were among first few cultures that adopted agriculture in early Holocene (about 9000 years ago; Pomeroy et al., 2019). This led to transition of lifestyle from hunter‐gatherer to farming, resulting in gradually decreasing physical stature and a lack of significant increase in lean mass. This in turn effectively decreased the metabolic capacity of modern‐day Asian Indians. In line with this, anthropometric evaluation of skeletons of Asian Indians spanning over the past 11,000 years showed gradually decreased stature‐adjusted lean mass. Thus, the modern Indians are the result of genotypic and phenotypic conditioning that occurred over thousands of years, which made them susceptible for conditions such as LD, irrespective of where they live now (Wells et al., 2016). This phenotype of Asian Indians has been well documented in recent studies (Bild et al., 2002; Kanaya et al., 2014; Bancks et al., 2021).

### 7.4 Diagnosis and management of lean diabetes

Unlike T1DM or T2DM, there are no specific guidelines for the diagnosis and clinical management of LD (Faraz et al., 2021; Kishore, 2022). Ironically, despite the rising prevalence of LD, a search of literature does not provide insights into the clinical management of LD. Diagnosis of LD is based on determination of BMI, and assessment of overweight and obesity independent of BMI as shown in Table 2, along with the determination of fasting and postprandial blood glucose levels. If possible, precise assessment of lean body mass vs. fat percentage using image technologies can be instituted (Table 2; Brunetti, 2007). Once the provisional diagnosis of LD is established, it is advisable to assess the insulin secreting capacity of the patients by biochemical assay for C‐peptide levels in plasma (Jones & Hattersley, 2013). Additional tests include determination of insulin sensitivity and/or insulin resistance with the technologies available in the clinics (Muniyappa et al., 2021).

Treatment of LD consists of two components, namely, to induce insulin secretion and to reduce insulin resistance. A South Asian Task Force comprising endocrinologists from India, Pakistan, Bangladesh, Nepal, Sri Lanka, Afghanistan, and the Maldives evaluated the use of GLP‐1 receptor agonists in the management of T2DM in South Asia, where LD is prevalent (Karla et al., 2019). In 2020 a clinical study was registered by researchers at the University of Leeds, United Kingdom to evaluate the combination therapy with Liraglutide (injectable GLP‐1 receptor agonist) and Pioglitazone (orally administered glitazone) in LD. The basis for this clinical trial is Liraglutide increases insulin secreting power of the pancreas, while Pioglitazone reduces resistance to insulin action Clinical Trials, NCT04657939 (n.d.).

### 7.5 Limitation of BMI as a measure of adiposity across populations

**FIGURE 9:**
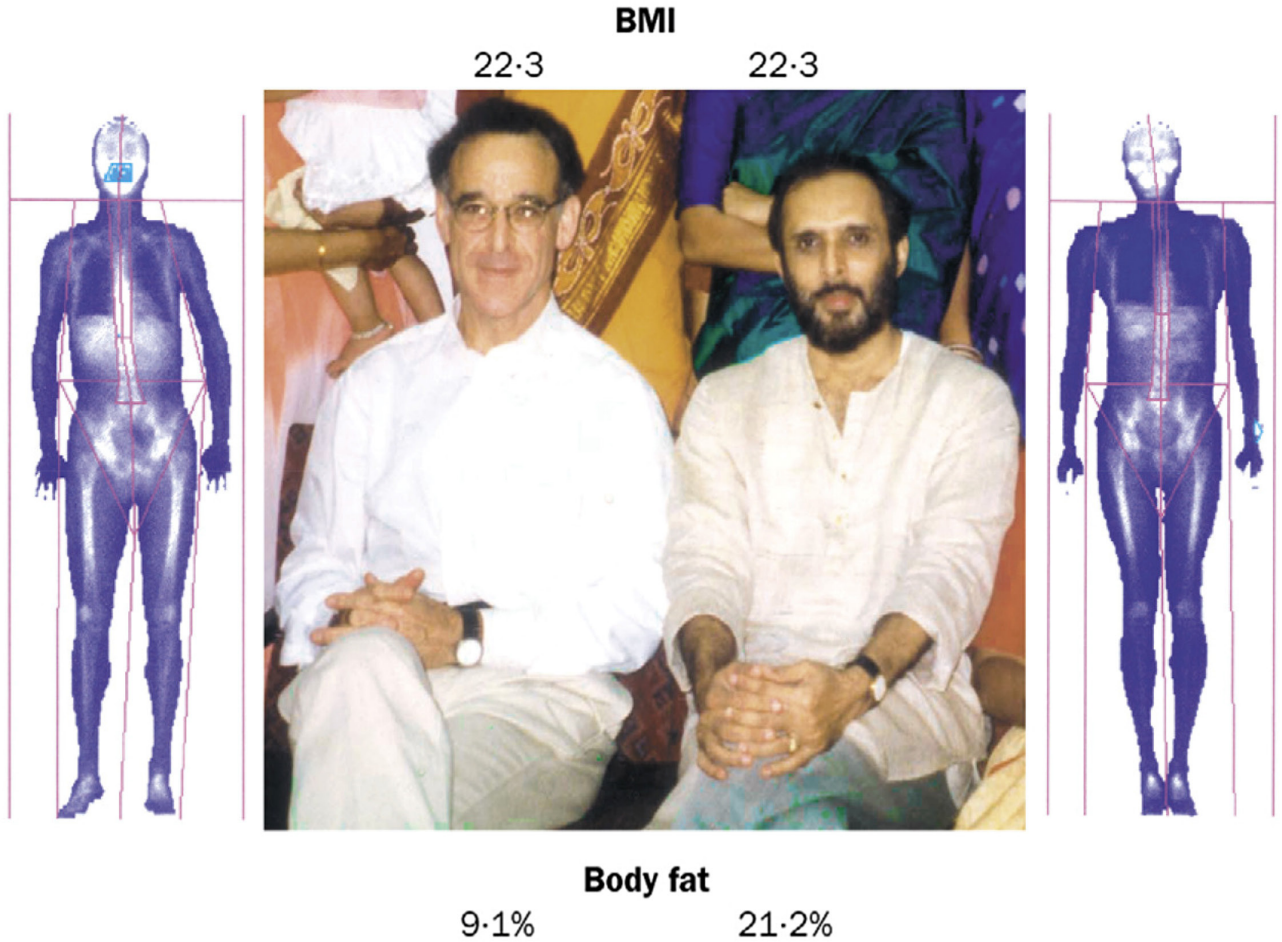
Limitations of BMI as a measure of adiposity in South Asians versus Caucasians. Two physician‐scientists, Dr. John S. Yudkin (left) and Dr. Chittaranjan S. Yajnik (right) share a near identical BMI (22.3), but as dual x‐ray absorptiometry imagery shows where the similarity ends. Dr. Yajnik has substantially more body fat (21.2%) than Dr. Yudkin (9.1%). Lifestyle may also be relevant. Dr. Yudkin runs marathons whereas Dr. Yajnik's main exercise is running to beat the closing doors of the elevator in the hospital every morning. The contribution of genes to such adiposity is yet to be determined, although the possible relevance of intrauterine undernutrition is supported by Dr. Yajnik's low birthweight. Thus, this image illustrates the role of both birthweight and lifestyle practices in body composition. Image and legend are reproduced with permission from: Yajnik CS, Yudkin JS, Lancet, 2004. Elsevier License No. 5812360591103 dated June 19, 2024.

The data and studies presented above convey that BMI is not a reliable measure of adiposity and cardiometabolic risk, especially when considering ethnic populations, such as Asian Indians. Chittaranjan S. Yajnik, M.D. of King Edward Memorial Hospital Research Center, Pune, India, and John S. Yudkin, FRCP of International Health and Medical Education Center, University College of London, United Kingdom, who did extensive research work on low birth weight and insulin resistance and diabetes in later life illustrated the limitation of BMI as a measure of adiposity by comparing the BMI and adiposity of their own bodies (Figure 9; Yajnik & Yudkin, 2004). This marked difference between the Asian Indians and Caucasians in body composition has been attributed to evolutionary factors as discussed above.

### 7.6 Thin‐obese paradox babies of India

**FIGURE 10:**
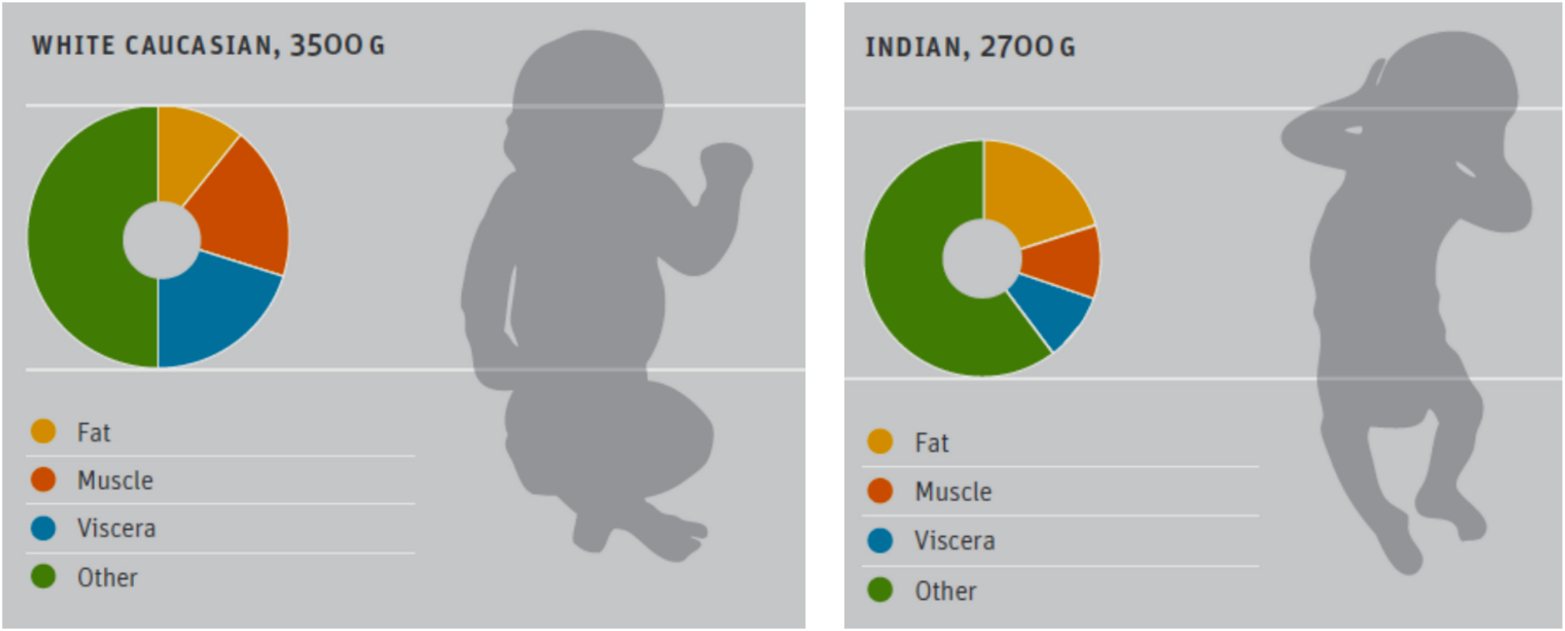
Thin‐fat Indian baby. A schematic diagram to compare the body composition of Indian and white caucasian babies. Indian babies were ~ 800 g lighter; muscle thin but more adipose tissue compared to the white babies. Reproduced from: Deshmukh et al., (2011) Sight Life Mag under Creative Commons Attribution 4.0 International License.

Because of the above evolutionary adaptation Asian Indian babies are born with a “thin‐fat phenotype,” comprising of thin muscles and relatively more adipose tissue (Deshmukh et al., 2011). This is not the case with Caucasian babies. A comparative study between the Indian versus British newborn babies confirmed the differences in phenotypes (Yajnik et al., 2003). As shown in Figure 10, the Indian newborns have low muscle mass and small abdominal viscera but preserved subscapular skinfold. This composition may persist postnatally thus predisposing to insulin resistance state. Because of this unique phenotype, Indian babies are metabolically programmed to develop T2DM early in adulthood, or even LD.

### 7.7 Maternal factors programming fetal cardiometabolic development

Although the existence of cross talk between adipose tissue and placenta in obese and gestational diabetes mellitus via exosomes has been known for a while (Jayabalan et al., 2017), recently, a novel possibility that an adiposity‐related maternal factor crossing the placenta to reprogram fetal cardiometabolic development pathways has been jointly reported by Robert J. Freishtat and his team at the Children's Research Institute, Washington DC and Chittaranjan Yajnik and his group at the King Edward Memorial Hospital, Pune, India (Kunte et al., 2024). They identified adipocyte‐derived exosomes that can cross the placenta, and their microRNA contents are predicted to alter developmental pathways of gene expression. Thus, their results suggest that adipocyte‐derived small extracellular vesicular (ADsEV) microRNAs in mothers are potential regulators of fetal adiposity. This joint project was funded by the US National Institutes of Health. Furthermore, the Pune Maternal Nutrition Study conducted by Dr. Chittaranjan S. Yajnik brought out the critical role played by the maternal diet and micronutrient status, and physical workload, during the pregnancy on the size of the newborn, development of insulin resistance, and cardiometabolic risk of the offspring in adulthood (Chittranjan, 2020; Fall, 2009; Tomar et al., 2015; Yajnik et al., 2003, 2008). This novel concept, which is a work in progress, has the potential to provide insights into programming fetal cardiometabolic development or risk by maternal factors. This in turn may prompt a paradigm shift in the global war against obesity from nutritional and lifestyle changes in adulthood to prenatal, perinatal, postnatal maternal and fetal/infant nutrition and care.

## 8 SUMMARY

Adiposity per se is not unhealthy, but its regional distribution, the type of fat expansion, and adaptation to excess caloric intake do matter for the ultimate pathophysiological roles. The existence of metabolically benign adipose tissue can largely explain the obesity paradox. The role of CRF in influencing obesity paradox is becoming obvious by allowing excess adiposity without risk. Further research into deciphering these potential possibilities is needed. Such studies may pave the way to develop novel molecular and/or imaging technologies for accurate phenotyping of patients to capture properly the trajectories of mortality in several disease conditions. Obviously, more clinical data with evaluation of current therapeutic methods are needed to manage LD.

## 9 CONCLUSION

**FIGURE 11:**
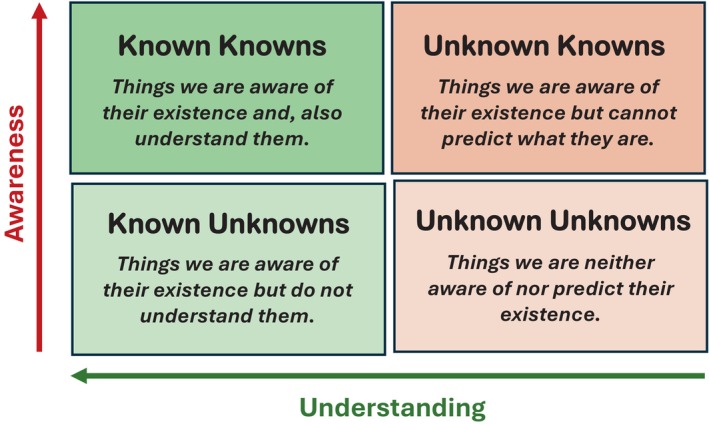
Modified rumsfeld knowledge and awareness matrix to obesity. The Rumsfeld knowledge matrix spans from “known knowns,” “known unknowns,” and “known unknowns” to “unknown unknowns.” Thus, it provides a matrix for awareness and Understanding. This model and process is very applicable to the science of obesity. The model presented here is modified by the author to suit the subject of obesity paradox.

The obesity paradox may appear as an artifact. But there is substantial evidence for the existence of it. We can feel its presence in the clinic, especially in relations to certain NCDs. Based on our current knowledge, we may not be able to provide an accurate mechanistic explanation for obesity paradox. There are several missing pieces of the jigsaw puzzle. Obviously, more controlled clinical studies supported by molecular and image technologies are needed to understand the phenomenon of obesity paradox at the cellular level. The clinical characteristics, pathophysiology, management, and mortality in LD pose a new challenge for practicing physicians. Understanding the emerging concept of role of maternal factors in programming cardiometabolic risk in fetal stage will ultimately open the doors for an effective control of prevalence of global obesity and NCDs. Thus, it appears obesity is a puzzle that fits into Rumsfeld Theorem (Figure 11). Application of AI may enhance our ability to understand obesity in its true sense and how elusive it can be. In this context, obesity paradox is like the Schrödinger's Cat—it is neither fact nor fiction. It is the sum of both until we analyze and understand it more thoroughly.

## FUNDING INFORMATION

No federal or industry support in preparing this review article.

## CONFLICT OF INTEREST STATEMENT

Part of this article was based on a CME lecture given by the author at the 39th Annual Convention of the American Association of Physicians of Indian Origin (AAPI) in 2021 and appeared as non‐peer reviewed synopsis of the lecture in the publication of AAPI. In addition to his academic appointment, the author is the Co‐Founder, President, Chief Executive Officer, and Chief Scientific Officer of ePurines, Inc., a therapeutic drug development startup in the University of Utah Research Park, Salt Lake City, Utah. ePurines is a spin out of the academic research with patents conducted by the author and his colleagues and is focusing on developing anti‐obesity drugs among others. However, there are no competing interests or industrial support in preparing or publishing this article.

## ETHICS STATEMENT

Not applicable.

## DISCLAIMER

No artificial intelligence (AI) or AI tools have been used in the preparation of this review article.

## Data Availability

Not applicable—no new data generated.
